# Research Advances for Virus-vectored Tuberculosis Vaccines and Latest Findings on Tuberculosis Vaccine Development

**DOI:** 10.3389/fimmu.2022.895020

**Published:** 2022-06-23

**Authors:** Zhidong Hu, Shui-Hua Lu, Douglas B. Lowrie, Xiao-Yong Fan

**Affiliations:** ^1^ Shanghai Public Health Clinical Center, Key Laboratory of Medical Molecular Virology of Ministry of Education (MOE)/Ministry of Health (MOH), Fudan University, Shanghai, China; ^2^ National Medical Center for Infectious Diseases of China, Shenzhen Third People Hospital, South Science & Technology University, Shenzhen, China

**Keywords:** tuberculosis, vaccine, viral vector, mucosal immunity, trained immunity

## Abstract

Tuberculosis (TB), caused by respiratory infection with *Mycobacterium tuberculosis*, remains a major global health threat. The only licensed TB vaccine, the one-hundred-year-old Bacille Calmette-Guérin has variable efficacy and often provides poor protection against adult pulmonary TB, the transmissible form of the disease. Thus, the lack of an optimal TB vaccine is one of the key barriers to TB control. Recently, the development of highly efficacious COVID-19 vaccines within one year accelerated the vaccine development process in human use, with the notable example of mRNA vaccines and adenovirus-vectored vaccines, and increased the public acceptance of the concept of the controlled human challenge model. In the TB vaccine field, recent progress also facilitated the deployment of an effective TB vaccine. In this review, we provide an update on the current virus-vectored TB vaccine pipeline and summarize the latest findings that might facilitate TB vaccine development. In detail, on the one hand, we provide a systematic literature review of the virus-vectored TB vaccines are in clinical trials, and other promising candidate vaccines at an earlier stage of development are being evaluated in preclinical animal models. These research sharply increase the likelihood of finding a more effective TB vaccine in the near future. On the other hand, we provide an update on the latest tools and concept that facilitating TB vaccine research development. We propose that a pre-requisite for successful development may be a better understanding of both the lung-resident memory T cell-mediated mucosal immunity and the trained immunity of phagocytic cells. Such knowledge could reveal novel targets and result in the innovative vaccine designs that may be needed for a quantum leap forward in vaccine efficacy. We also summarized the research on controlled human infection and ultra-low-dose aerosol infection murine models, which may provide more realistic assessments of vaccine utility at earlier stages. In addition, we believe that the success in the ongoing efforts to identify correlates of protection would be a game-changer for streamlining the triage of multiple next-generation TB vaccine candidates. Thus, with more advanced knowledge of TB vaccine research, we remain hopeful that a more effective TB vaccine will eventually be developed in the near future.

## 1 Introduction

Among the top 10 leading causes of death worldwide and the leading cause of death by a bacterial infection, tuberculosis (TB) caused 1.5 million deaths in 2020 (1). According to the World Health Organization (WHO), a quarter of the world population is infected with *Mycobacterium tuberculosis* (*Mtb*), the cause of TB (1). The Coronavirus Disease 2019 (COVID-19) pandemic reduced access to TB diagnosis and treatment and led to an increase in TB deaths in 2020, the first time during the past decade (1). Ambitious targets for TB control have been set by WHO. The “End TB Strategy” defined milestones and targets that aim to reduce TB incidence by 90% and deaths by 95% by 2035 compared with 2015 (2). However, progress has been slow and the 2020 milestone was far from reached (1). The lack of an optimal TB vaccine is regarded as one of the key barriers for TB control, thus, WHO timelines to control the global TB epidemic require a vaccine that is more effective, particularly in adolescents and adults. In this review, we focus on the history of research and the developmental progress of virus-vectored TB vaccines that are in clinical trials, those that are in pre-clinical animal research, and the latest ancillary findings facilitating TB vaccine research and development.

## 2 One-Hundred-Year-Old BCG: Successes and Failures

*Mycobacterium bovis* Bacille Calmette-Guérin (BCG), a vaccine based on attenuation of a bacterium naturally causing TB in cattle, is the only licensed TB vaccine up to now. BCG was first administered to the newborn infant of a woman with TB in France in 1921, neither adverse effects nor disease developed in the subsequent 5.5 years (3). In the next several years, thousands of children in families with a history of TB received BCG at the Pasteur Institute (4). In 1928, the intradermal route was found to be more reliable compared with the oral route, and this route continues to be used today (5). In the same year, the League of Nations (the predecessor of the United Nations) declared BCG to be safe for use. Although the “Lübeck disaster”, in which 72 newborn babies died from TB after BCG vaccination due to contamination with a virulent strain of *Mtb* (6), hindered the public acceptance of BCG for a long time, a resurgence of TB during World War II led to widespread BCG inoculation and public confidence in its safety was regained (7). In 1974, the WHO created the “Expanded Programme on Immunization”, to ensure that mothers and children have universal access to routinely recommended neonatal vaccines, and this resulted in more than 4 billion BCG vaccinations being administered to date (8). The vaccine has probably been administered to more humans than any other vaccine. Two-thirds of those countries giving BCG vaccination are estimated to have more than 90% coverage (9) and the vaccine is still the gold standard against which new candidates are compared.

The widespread use of the BCG vaccine in infants continues, primarily because it offers protection against the aggressive childhood forms of the disease: meningeal and miliary TB (10). However, for pulmonary TB prevention in adults, clinical trials have estimated its vaccine efficacy to range from 0% in south India to 80% in the UK (11). There are many hypotheses to explain this wide variation, including age at vaccination (12, 13), exposure to environmental mycobacteria (14), gender (15), risk of TB in the study population (16), etc. However, the proposed causes of variation often remain speculative and the basis is likely to be multifactorial.

The failure of TB control indicates that BCG is insufficient. Strategies to improve TB vaccination mainly address one of two approaches: optimization of the current BCG vaccine or development of novel vaccines such as subunit, vectored, and live attenuated vaccines. In the optimization of BCG we include BCG re-vaccination, change of inoculation route, and recombinant BCG construction. Although WHO does not recommend BCG re-vaccination due to a lack of proven efficacy of repeat doses for protection against TB, the most recently completed clinical trials showed BCG re-vaccination had an efficacy of 45.4% in primary *Mtb* infection prevention, which was defined immunologically by QuantiFERON-TB Gold In-tube assay conversion (17). However, the side effects of BCG re-vaccination hinder its application in humans with immune disorders. Although intravenous BCG immunization was consigned to the history books, this approach was recently re-evaluated in the non-human primate model of TB, in which nine out of ten intravenous BCG-vaccinated macaques showed slight or even no signs of TB disease post *Mtb* infection (18). However, safety concerns will impede application in humans. One of the most promising TB vaccines may provide an alternative. The genetically modified BCG-based vaccine VPM1002, in which the gene encoding urease C was replaced by the listeriolysin encoding gene from *Listeria monocytogenes*, showed the potential to replace the current BCG vaccine and is now undergoing three phase III clinical efficacy trials (19).

Besides the optimization of the current BCG vaccine, another approach is to utilize novel TB vaccines as a booster of the BCG vaccine, since most adults who acquire TB worldwide today were BCG-vaccinated as neonates. In the past decades, several viral vector-based vaccines and protein-adjuvant vaccines were designed to enhance BCG-primed immune protection. In this review, we focus on the research and development of viral vector-based TB vaccines.

## 3 Recombinant Virus-vectored TB Vaccines

Viruses provide some of the most widely used vaccine vectors. Recombinant virus-vectored vaccines are capable of inducing robust immune responses by mimicking the processes of pathogens invading the organism and resulting in the formation of long-lasting immune memory. Basically, most viral vaccine vectors have the following advantages: 1) they can accommodate genes encoding large antigenic fragments; 2) they have stable exogenous gene expression efficiency; 3) they can induce high levels of both cellular and humoral immune responses; 4) the immune responses induced by the vector itself have the potential to augment the antigen-specific immune memory to some extent; 5) they do not always require the use of adjuvants; 6) they are easy to manipulate and culture; 7) the use of attenuated or replication-deficient viruses with a clear mechanism of infection provides a strong safety profile; 8) strong immune memory can generally be induced by a single immunization, and repeated vaccinations might not be required (20–24). The major disadvantages of viral-vectored vaccines includes: 1) the pre-existing neutralizing antibodies against the vector might limiting its application in humans; 2) the host-induced anti-vector immunity might limit the booster vaccination strategies; 3) some viral vectors are not appropriate for use in immunocompromised individuals.

Although it was well illustrated that Th1 CD4^+^ T cell responses dominated anti-TB immune protection, *Mtb* can survive intracellularly for a long time after primary infection, the induction of immune responses that include high levels of cytotoxic T lymphocyte is also crucial to the clearance of intracellular *Mtb* (25–27). Cytotoxic T cells are prominent in the immune response to viruses and viruses accordingly provide one of the most widely used vector formats in the field of TB vaccines.

### 3.1 Mechanisms of Immune Protection Afforded by Virus-vectored Vaccines

The mechanisms of immune protection vary depending on the nature of the induction stimulus. Overall, recombinant viral-vectored vaccines carrying exogenous antigen fragments are able to invade host cells by using intrinsic viral mechanisms and undergo massive intracellular replication. The intracellular products and those secreted extracellularly induce cellular and humoral immune responses, respectively. The commonly-used attenuated or replication-deficient viruses are rapidly cleared after the host’s immune response is activated, while the antigen-specific immune cells are gradually transformed into memory cells that can remain for a long time. In addition, recombinant viral-vectored vaccines are able to induce a strong co-stimulatory molecular signaling and the formation of an inflammatory microenvironment, which together act as signals 2 and 3 of the T-cell/B-cell response pathways to enhance the host’s antigen-specific adaptive immunity.

### 3.2 Brief Introduction of the Widely Used Viral Vectors

The poxviruses are among the most widely studied viral vectors. They constitute a group of double-stranded DNA viruses that is divided into 2 subfamilies and 12 genera, among which, Orthopoxvirus, Molluscipoxvirus, Parapoxvirus, and Yatapoxvirus are known to infect humans (28, 29). Poxviruses of different genera infect different animals to cause different diseases and vaccinia virus, belonging to the genus Orthopoxvirus, although not fully non-pathogenic in human, has been a highly effective “live” vaccine against the smallpox epidemics that once ravaged humans (30). For safety reasons, most studies have chosen to use replication-deficient versions of poxviruses in vaccine vector development. The reduced immunogenicity consequent upon reduced replication can be offset by genetic modifications to knock-down molecules used by the virus to attenuate immune responses and by expression of immunostimulatory molecules in addition to the target antigens (31, 32). The types of genes encoded to enhance immune responses include: type I and type II interferons, genes regulating cytokines and chemokines, apoptosis and immunosuppression related molecules, antigen presentation signaling pathway molecules, etc. (33–35) Notable among poxvirus vaccine vectors are four strains of replication-deficient poxviruses, including modified vaccinia virus Ankara (MVA) (36), NYVAC derived from Copenhagen strain (37), ALVAC modified from canary poxvirus (38), avian poxvirus TROVAC (38), and another attenuated vaccinia strain, namely Chinese Tiantan strain poxvirus (39). The recombinant vaccine MVA85A, also known as AERAS-485, expresses *Mtb* immunodominant antigen Ag85A and was the first new TB vaccine to complete phase IIb clinical trials (40). It induced strong immune responses among Th1 and Th17 CD4^+^ T cells, in addition to moderate CD8^+^ T cell responses (41).

Adenovirus (Ad) is another widely used vaccine vector. About 50 human adenovirus (AdHu) serotypes have been identified, of which AdHu5 and AdHu35 are the two most widely used subtypes. Two human adenovirus-based recombinant vaccines against TB, AdAg85A (also known as AdHu5Ag85A) and AERAS-402 (also known as Crucell Ad35), in addition to ChAdOx1.85A, based on a chimpanzee adenovirus vector, are capable of inducing a strong CD8^+^ T-cell immune response in addition to high levels of Th1-type CD4^+^ T-cell immune response.

Other viral vectors including influenza virus, cytomegalovirus (CMV), Sendai virus (SeV), lentivirus, vesicular stomatitis virus (VSV), have also been applied to TB vaccine studies. Although these viruses infect cells by different mechanisms, most of them can induce high levels of antigen-specific Th1 CD4^+^ and CD8^+^ T cell immune responses as TB vaccine vectors. Among them, SeV85AB, a recombinant SeV-vectored vaccine expressing Ag85A and Ag85B, was the first viral-vectored TB vaccine found able to induce high levels of lung tissue-resident memory T cells (T_RM_)-mediated immune protection (42) and provided a new research direction for TB vaccines.

### 3.3 Brief History of Research and Development of Virus-vectored TB Vaccines

The recombinant poxvirus-vectored vaccine MVA85A was the first of the new TB vaccines to complete phase IIb clinical trials (40), but there are several other recombinant viral-vectored vaccines against TB in clinical and preclinical phases of evaluation. Herein, we give a brief overview of the history and the latest discoveries in the field.

#### 3.3.1 MVA85A/AERAS-485

The MVA85A vaccine, which expresses the *Mtb* immunodominant antigen Ag85A, was developed by the University of Oxford in 2001, researchers found that intradermal (i.d.) or intramuscular (i.m.) immunization with this vaccine was able to induce a high level of antigen-specific immune response and protective immunity against *Mtb* challenge by decreasing the bacterial loads in organs in mouse models (43–45); subsequently, the vaccine-induced protection was further validated in other animal models such as guinea pigs (46), cattle (47) and rhesus monkeys (48). In the first phase I clinical trial, published in 2004, the vaccine was inoculated i.d. and induced a strong specific T-cell immune response in adults with or without a BCG immunization history (49). In 2010, the immunogenicity and safety of this i.d. vaccine were further confirmed in children and adolescents (50). Two phase I clinical trials completed in 2012 and 2013, were conducted to optimize the immunization dose (51) and route of administration (i.m. and i.d.) (52). During this period, from 2009 to 2011, researchers recruited 2,797 BCG-immunized infants between 4 and 6 months of age and i.d. administered either MVA85A or placebo and followed them for 19 to 28 months to complete the first phase IIb clinical trial (40). Although the safety of the vaccine was strongly demonstrated, the vaccine only induced weak antigen-specific immune responses and was not protective against TB disease (40), similar with the results that MVA85A did not reduce the bacterial burden of BCG-prime mice (53). Thus, the first viral-vectored TB vaccine clinical trial was declared a failure. In a proof-of-concept phase II trial, the number of immunization times was increased to two shots in a population of HIV-infected patients, but the efficacy, which was defined by QuantiFERON-TB Gold In-Tube conversion, remained poor (54). Nonetheless, researchers have not given up their efforts. A phase I clinical trial study in 2014 reported validation of the safety of mucosal immunization with MVA85A (55), and in 2019 a phase I clinical trial showed that an aerosol prime-intradermal boost regime was well-tolerated and induced potent antigen-specific mucosal and systemic immune responses (56). Additionally, i.m. inoculation was tested in a phase II clinical trial (NCT02178748) that indicated that a change in the route of administration may be a way to improve the vaccine’s protective efficacy. In 2021 a phase I trial showed that MVA85A delivered by aerosol was safe in UK adults with latent TB infection (57). Utility in a potential niche application was indicated in a phase II clinical trial that showed MVA85A vaccination in HIV-exposed newborns might be used to avoid the potential risk of BCG disease in this population (58).

Pre-clinical studies had indicated that the vaccine might work best as a booster in combination with other vaccines. To test this clinically, the vaccine was combined with the recombinant adenovirus-vectored vaccine AERAS-402 (59) with ChAdOx1.85A (currently in a phase II trial, NCT03681860) (60), with the recombinant avian poxvirus-vectored vaccine FP85A (61), or the protein adjuvant vaccine IMX313 (62), all of which had all been validated in phase I clinical trials.

Besides MVA85A, the potential of several other MVA-based recombinant TB vaccines has been indicated. For example, a multiphasic vaccine expressing 14 antigens representative of the three phases of TB infection (active, latent, and resuscitation) was subcutaneously (s.c.) immunized and induced potent multifunctional cell-mediated immunity in mice and rhesus macaque models (63). A recombinant MVA expressing α-crystallin by using i.d. route enhanced BCG-induced protection against *Mtb* infection in guinea pigs (64).

#### 3.3.2 AdAg85A

Recombinant adenovirus vectors are widely used in the field of TB vaccine research. At least three vaccines are currently moving forward in clinical trials: AdAg85A based on AdHu5 (65, 66), AERAS-402 based on AdHu35 (67–73), and ChAdOx1.85A (60) based on a chimpanzee adenovirus vector, which would be discussed in detail below.

Recombinant AdHu5 vectored vaccine AdAg85A, was developed by McMaster University and published in 2004 (74). Similar to the MVA85A vaccine study, the safety, immunogenicity, and protective efficacy of the AdAg85A vaccine by using the aerosol and i.m. route were validated in animal models including mice (75–77), guinea pigs (78), cattle (79), goats (80), and rhesus macaques (81) before entering clinical trials. In the first clinical trial in 2013, AdAg85A was i.m. administrated in BCG-naïve and previously BCG-immunized healthy adults, and strong antigen-specific CD4^+^ and CD8^+^ T cell responses were observed (65). Most recently, in 2022, a phase Ib trial showed that aerosol delivery of AdAg85A was also safe and well-tolerated in previously BCG-vaccinated adults (66). A potential disadvantage of the vaccine is that substantial levels of anti-AdHu5 antibodies tend to be preexisting in humans, although the inventors of the vaccine demonstrated that AdHu5 antibodies do not affect the safety and immunogenicity of AdAg85A (65). However, a clinical trial of an AdHu5-based HIV vaccine was terminated due to the discovery that vaccinated subjects who had high titers of antibodies against adenovirus tended to have a higher incidence of HIV acquisition than those without anti-adenovirus antibodies in 2007 (82). Consequently, the role of preexisting vector-specific antibody responses remains controversial and there is currently an international preference for the use of AdHu35, the antibodies of which are largely absent from human serum. In addition, considering low sero-reactivity was observed in chimpanzee- and simian-derived adenoviral vectors compared with human-derived vectors in humans (83), several recombinant chimpanzee adenovirus-vectored TB vaccines were constructed, which will be described below. However, through a mouse model, the magnitude, quality and protective capacity of CD8^+^ T cells elicited using simian immunodeficiency virus Gag as the target antigen were compared, AdHu5 and AdCh3 vectors conferred the best efficacy (83, 84). These studies added a layer of complexity to balancing safety and vaccine efficacy in choosing adenovirus vectors.

#### 3.3.3 AERAS-402/Crucell Ad35

The AdHu35-based recombinant vaccine, AERAS-402, expressing Ag85A, Ag85B, and TB10.4 was developed jointly by Crucell and the Aeras organization. In 2007, it was shown that this vaccine was i.m. immunized and was able to induce a strong T-cell immune response and a strong immune-protective effect against *Mtb* in a mouse model (85). The protection afforded by AERAS-402 singly or in combination with other vaccines was also validated in rhesus macaques (86–88). In 2010, the i.m. vaccine’s safety and immunogenicity were confirmed in healthy adults in a phase I clinical trial (67). In subsequent years, through several phase I and phase II clinical trials, researchers have expanded the potential target population to include healthy infants previously vaccinated with BCG (68), healthy adults immunized with BCG (69), adults with active or previous TB (70), latently infected populations (71), and HIV-infected patients (72). A two-dose i.m. regimen was also evaluated in BCG-vaccinated adults in phase I clinical trial in 2021 (73). Several AERAS-402-based phase II clinical trials targeting different populations, including adults treated for pulmonary TB, HIV-infected/BCG-vaccinated adults, and BCG-vaccinated healthy infants, have been completed (NCT02414828, NCT01017536, and NCT01198366). The safety of i.m. AERAS-402 was confirmed through these trials.

#### 3.3.4 ChAdOx1.85A

To minimize any effects of preexisting anti-adenovirus antibodies in humans, researchers have developed the recombinant replication-deficient chimpanzee adenovirus-vectored vaccine ChAdOx1.85A. Its i.m. immunization is capable of inducing high levels of cellular immune response in BCG-primed mice and showing protective efficacy against *Mtb* infection in combination with MVA85A (89, 90). In 2020, a phase I clinical trial demonstrated that a ChAdOx1.85A i.m.-MVA85A i.m. vaccination regimen was well tolerated and immunogenic in healthy UK adults (60). As mentioned above, this vaccine strategy is now in a phase II clinical trial (NCT03681860).

Intranasally (i.n.) immunization with a recombinant ChAdOx1 vaccine expressing Rv1039c (PPE15) instead of Ag85A conferred better protection than ChAdOx1.85A in a murine model, meriting further evaluation in clinical trials (91). Similarly, a recombinant chimpanzee adenovirus-68-vectored vaccine expressing Ag85A, namely AdCh68Ag85A, was i.n. immunized and found to be superior to AdAg85A in the induction of T-cell responses and protection against *Mtb* infection in mice that had previously been exposed to human adenovirus (92). In addition, there is evidence that this vaccine could be used as a therapeutic vaccine: Immunotherapy with a single-dose respiratory mucosal but not parenteral application of AdCh68Ag85A as an adjunct to antibiotic therapy accelerated pulmonary *Mtb* clearance, limited lung pathology, and restricted disease in mice (93).

#### 3.3.5 TB/FLU-04L

In 2006, a recombinant influenza virus-vectored vaccine expressing ESAT-6 was shown to be able to induce a high level of Th1 CD4^+^ T cell immune response with two i.n. injections in a mouse model (94). The protective efficacy of the vaccine was confirmed in mice and guinea pigs (95). This vaccine, named TB/FLU-04L, was aerosol immunized and completed a phase I clinical trial in 2015, but no study results have been published (NCT02501421). According to WHO reports in 2017 (96), a phase IIa clinical trial is being conducted in patients with latent TB infection. Besides TB/FLU-04L, another recombinant influenza virus-based vaccine expressing the dominant peptides of Ag85B was constructed, and robust T_RM_ responses and protective efficacy were observed in a murine model by using i.n. route (97).

#### 3.3.6 MCMV85A and RhCMV/TB

In 2014, a recombinant murine CMV-vectored vaccine MCMV85A expressing Ag85A was developed by the University of Oxford. This vaccine was inoculated intraperitoneal (i.p.) or intravascular (i.v.), and activated NK cells to provide early nonspecific protection against *Mtb* infection, which was further potentiated by a weak 85A-specific T cell response in a murine model (98). In 2018, a rhesus monkey CMV vector vaccine RhCMV/TB was described that encoded nine proteins from three phases of *Mtb* infection: acute (Ag85A, Ag85B, ESAT-6), latency (Rv1733, Rv2626, Rv3407), and resuscitation (RpfA, RpfC, RpfD). Two doses of s.c. RhCMV/TB induced high levels of specific CD4^+^ and CD8^+^ T cell immune responses, and provided long-lasting vaccine-mediated immune control after highly pathogenic *Mtb* strains challenge one year after immunization in rhesus macaques, in which 41% animals showed no TB disease evaluated by computed tomography scans or necropsy (99). However, although human CMV infection only causes asymptomatic infection in the immunocompetent population, CMV is highly species-specific and systemic disease with severe complications and high mortality rate might be occurred in immunocompromised individuals (100, 101). Moreover, epidemiological studies have identified the increased human CMV infection is an important risk factor for active TB disease and latent TB infection, which was found to be associated with the magnitude of IgG, enhanced CMV-driven T-cell activation, systemic inflammation, and immune dysregulation (102, 103). Thus, more animal and clinical studies are warranted to better understand CMV-vectored immunity, to ensure its safe translation to humans, especially in active TB patients and individuals with latent TB infection.

#### 3.3.7 SeV85AB

SeV85AB, a recombinant SeV-vectored vaccine, is the first application of a SeV vector to the TB vaccine development and it expresses *Mtb* immunodominant antigens Ag85A and Ag85B and inherently has a high safety profile. Being based upon an RNA virus, the SeV vector has no risk of integration with the human genome. Furthermore, in contrast to respiratory pathogens such as the influenza virus, the SeV does not cause human disease and there are very low antibody levels present. In 2017, using a mouse model, we validated its immunogenicity and protective efficacy against *Mtb* infection in mice and demonstrated the establishment of a high level of T_RM_-mediated immune response in mucosal tissues by using i.n. route (42). Such memory cells can establish the first line of defense in the lung against *Mtb* invasion in the early phase of infection. In contrast, BCG vaccination usually produces a response of memory T cells in the circulatory system only after several weeks of infection. Therefore, this vaccine may be used to optimize the systemic BCG-induced immune protection against *Mtb* infection (104). This immunization strategy was further optimized in combination with recombinant DNA vaccines for improved protective efficacy (105).

#### 3.3.8 Other Viral-vectored Vaccines

Several other promising viral vectors are being explored as candidates for TB vaccine construction. In 2008, Hamamatsu University School of Medicine constructed a lentiviral vector vaccine expressing MPT51 of *Mtb*. This construct enhanced the antigen presentation efficiency of dendritic cells, and intratracheal (i.t.) immunization of mice was able to induce a CD8^+^ T-cell immune response at the lung site and protection against *Mtb* infection (106). Recently, several other lentiviral vector-based TB vaccines have been developed, but they are at early animal model phases of investigation (107–110). Lentiviral vectors have been successfully used in the clinical trials of patients with advanced leukemia and other gene immunotherapy research (111, 112). In most of these TB vaccine studies described above, self-inactivation or non-integrating vector systems were chosen to get safe vaccines.

In 2008, researchers at McMaster University constructed a recombinant VSV-vectored vaccine VSVAg85A that expressed Ag85A and was able to induce an antigen-specific immune response and protection against *Mtb* infection but only for a short duration by using i.n. or i.m. routes. Combining the vaccination with AdAg85A in a prime-boost immunization strategy improved the protective efficacy (113); VSV-based boosting resulted in inferior protection compared with adenovirus-based boosting, and this was associated with differentially imprinted innate phagocytes at the mucosal site of immunization (114). Besides VSVAg85A, another VSV-based TB vaccine expressing Rv2660c, Rv3615c, and Mtb10.4 has generated antigen-specific T cell responses and immune protection in a BCG challenge murine model by using i.n. route (115, 116).

The use of a combination of vectors expressing the same antigen in order to enhance responses is a recurring theme. As mentioned in Section 3.3.1 above, a recombinant fowlpox virus FP9 that expressed Ag85A (FP9.Ag85A or FP85A) and boosted BCG/MVA85A-induced protective immunity in guinea pigs (46) also boosted immune responses to MVA85A in a clinical phase I trial in 2013 (61).

In 2014, a recombinant human parainfluenza type 2 virus-vectored vaccine expressing Ag85B, rhPIV2-Ag85B, was developed by the National Institute for Biomedical Innovation in Japan (117). This i.n. vaccine was able to induce a T-cell immune response and immune protection in a murine model that was subsequently found to be associated with induction of bronchus-associated lymphoid tissue (118). Similarly, a parainfluenza virus 5 vector expressing Ag85A and Ag85B has also shown immunogenicity and protective efficacy in a murine infection model by using i.n. route (119).

In 2020, different prime-boost strategies using the chimpanzee Ad3 (ChAd3) and MVA vectors expressing Ag85B, ESAT-6, Rv1733, Rv2626, and RpfD, were evaluated for immunogenicity and protective efficacy in highly susceptible rhesus macaques through different inoculation routes such as i.m., i.d., and aerosol. However, although specific immune responses were induced, none of these vaccine strategies conferred a protective effect compared to non-vaccinated controls (120).

To be noted, most of these studies used *Mtb* immunodominant antigens such as Ag85A, Ag85B, TB10.4, ESAT-6, etc., which were chosen based on their expression levels in *Mtb* and IFN-γ-inducing ability. However, the failure of MVA85A in its first phase IIb clinical trial suggest other antigens should be selected to construct a more effective vaccine. Recently, an unbiased immunopeptidomics pipeline for identifying novel antigens presented by MHC was developed, in which MHC I and MHC II complexes from BCG-infected THP-1 macrophages were immunoprecipitated and analyzed by liquid chromatography tandem mass spectrometry (121). Thus, identifying more efficient antigens by novel assays is also important in virus-vectored TB vaccine development.

**Table 1** summarizes the viral vectored TB vaccine candidates that are currently in clinical trials, and **Table 2** summarizes the candidates that are currently in preclinical animal model phases. These studies of novel virus-vectored TB vaccines have been successful in developing a number of candidates that have entered the TB vaccine pipeline (https://www.tbvi.eu/what-we-do/pipeline-of-vaccines/) and are at different stages of clinical trials in humans. This may lead to newly licensed vaccines capable of replacing/supplementing the current BCG vaccine and even conferring therapeutic benefit in patients with active/latent TB.

**Table 1:** Viral vectored TB vaccine candidates that are currently in clinical trials.

Candidate vaccines	Vectors	Antigens	Populations/animals	Clinical trial phases	Clinical trialstatus	Sponsors/inventors	References/clinical trial registry numbers
MVA85A i.d.	MVA	Ag85A	BCG-vaccinated healthy infants	IIb	Completed	Aeras, University of Oxford	37/NCT00953927
MVA85A i.d.	MVA	Ag85A	Adults infected with HIV-1	II	Completed	Aeras, University of Oxford	50/NCT01151189
MVA85A i.m.	MVA	Ag85A	BCG-vaccinated healthy adolescents	II	Completed	University of Oxford	NCT02178748
ChAdOx1.85A i.m. -MVA85A i.m.	ChAdOx1/MVA	Ag85A	Healthy adults and adolescents	II	Active, not recruiting	University of Oxford	NCT03681860
AERAS-402 i.m.	AdHu35	Ag85A, Ag85B, TB10.4	Adults treated for pulmonary TB	II	Completed	Aeras, Crucell	NCT02414828
AERAS-402 i.m.	AdHu35	Ag85A, Ag85B, TB10.4	HIV-infected, BCG-vaccinated adults	II	Completed	Aeras, Crucell	NCT01017536
AERAS-402 i.m.	AdHu35	Ag85A, Ag85B, TB10.4	BCG-vaccinated healthy infants	II	Completed	Aeras, Crucell	NCT01198366
TB/FLU-04L aerosol	FLU-04L	ESAT-6	BCG-vaccinated healthy adults	IIa	Unknown	Research Institute for Biological Safety Problems	NCT02501421 and unkown
AdAg85A aerosol	AdHu5	Ag85A	BCG-vaccinated healthy adults	Ib	Completed	McMaster University	70/NCT02337270
AdAg85A i.m.	AdHu5	Ag85A	BCG-naïve and -vaccinated healthy adults	I	Terminated	McMaster University	69/NCT00800670
MVA85A aerosol	MVA	Ag85A	BCG-vaccinated healthy adults	I	Completed	University of Oxford	51/NCT01497769
MVA85A aerosol-MVA85A i.d.	MVA	Ag85A	BCG-vaccinated healthy adults	I	Completed	University of Oxford	52/NCT01954563
MVA85A aerosol	MVA	Ag85A	Healthy adults with latent TB infection	I	Completed	University of Oxford, University of Birmingham	53/NCT02532036
AERAS-402 i.m.-MVA85A i.d.	AdHu35/MVA	Ag85A	BCG-vaccinated healthy adults	I	Completed	University of Oxford, Aeras, Crucell	55/NCT01683773
MVA85A i.d.-FP85A i.d.	MVA/FP9	Ag85A	BCG-vaccinated healthy adults	I	Completed	University of Oxford	57/NCT00653770
MVA85A i.d.-IMX313 i.d.	MVA/nanoparticle	Ag85A	BCG-vaccinated healthy adults	I	Completed	University of Oxford	58/NCT01879163

i.d., intradermal; i.m., intramuscular.

**Table 2:** Viral vectored TB vaccine candidates that are currently in preclinical animal model phases.

Candidate vaccines	Vectors	Antigens	Animals	Protective efficacy^a^	Route/dose of *Mtb* challenge	Sponsors/inventors	References
RhCMV/TB s.c.	RhCMV	Ag85A, Ag85B, ESAT-6, Rv1733, Rv2626, Rv3407, RpfA, RpfC, RpfD	Rhesus macaques	~2^b^	i.b., 25/10 CFU	Oregon Health and Science University	(99)
MVA multiphasic s.c.	MVA	RpfB, RpfD, Ag85B, TB10.4, ESAT-6, Rv2029, Rv2626, Rv1733, Rv0111, Rv0569, Rv1813, Rv3407, Rv3478, Rv1807	Rhesus macaques	N/A	N/A	Transgene, Advanced BioScience Laboratories	(63)
ChAd3-5Ag aerosol/i.m. prime, MVA-5Ag aerosol/i.d. boost	ChAd3-MVA	Ag85B, ESAT-6, Rv1733, Rv2626, and RpfD	Rhesus macaques	NS	i.b., ~15 CFU	Biomedical Primate Research Center	(120)
rMVA.acr i.d.	MVA	α-crystallin	Guinea pigs	1.27^c^	Aerosol, 5-10 CFU	University of Delhi South Campus	(64)
SeV85AB i.n.	SeV	Ag85A, Ag85B	Mice	~0.8	Aerosol, ~100 CFU	Shanghai Public Health Clinical Center, ID Pharma	(42, 104, 105)
ChAdOx1.Rv1039c i.n.	ChAdOx1	Rv1039c	Mice	~1	Aerosol, 50-100 CFU	University of Oxford	(91)
AdCh68Ag85A i.n.	AdCh68	Ag85A	Mice	~0.7	Aerosol, ~100 CFU	McMaster University	(92, 93)
PR8.p25 i.n.	H1N1 PR8	Ag85B	Mice	~0.5	Aerosol, ~100 CFU	The University of Sydney	(97)
MCMV85A i.v.	MCMV	Ag85A	Mice	~0.6	i.n., ~200 CFU	University of Oxford, Ludwig Maximilians University	(98)
MPT51 lentivirus i.t.	Lentivirus	MPT51	Mice	~1	i.t., 1.2×10^4^ CFU	Hamamatsu University School of Medicine	(106)
LAR f.p.	Lentivirus	Ag85B, Rv3425	Mice	~1^c^	i.v., 1.2×10^6^ CFU	Fudan University	(107)
LV vF/85A s.c./i.n.	Lentivirus	Ag85A	Mice	NS	i.n., 5×10^6^ CFU^d^	University College London	(108)
A3-Len f.p.	Lentivirus	Ag85B, Rv3425	Mice	~0.3	i.v., 6.8×10^5^ CFU	Fudan University, Institute Pasteur of Shanghai	(109)
LV-AEG/SVGmu f.p.	Lentivirus	Ag85A, ESAT-6	Mice	N/A	N/A	Pasteur Institute of Iran	(110)
VSVAg85A i.n./i.m.	VSV	Ag85A	Mice	~0.6/0.1	i.n., ~100 CFU	McMaster University	(113, 114)
VSV-846 i.n.	VSV	Rv2660c, Rv3615c, Mtb10.4	Mice	~1.5	i.n., 1×10^7^ CFU^d^	Soochow University	(115, 116)
rhPIV2-Ag85B i.n.	hPIV2	Ag85B	Mice	~1.9	Aerosol, ~50 CFU	National Institute for Biomedical Innovation	(117, 118)
PIV5-85A/PIV5-85B i.n.	hPIV5	Ag85A/Ag85B	Mice	~1.2/0.4	Aerosol, 50-100 CFU	University of Georgia College of Veterinary Medicine	(119)

a Bacterial load log reduction compared with vector-immunized/non-immunized animals in the lung.

b Log reduction in the density of culturable Mtb (CFU/g) in all lung-draining lymph nodes.

c Bacterial load log reduction of BCG prime-candidate vaccine boost group compared with BCG immunization group.

d BCG infection.

f.p., foot pad; i.b., intrabronchial; i.d, intradermal; i.m., intramuscular; i.n., intranasal; i.p., intraperitoneal; i.t., intratracheal; i.v., intravascular; s.c., subcutaneous; N/A, not available; NS, not significant.

## 4 Latest Findings That Facilitate TB Vaccine Research Development

Over the past two decades, huge progress has been achieved in the field of TB vaccine development and more than a dozen candidate vaccines including viral-vectored vaccines are in clinical trials now. However, several knowledge gaps and challenges to the successful development of a universally effective TB vaccine remain. Here we summarize the latest findings facilitating TB vaccine research development.

### 4.1 Controlled Human Infection Model

The immunogenicity and protective efficacy of the TB vaccines that have entered clinical trials were first repeatedly validated in animal models such as mice, guinea pigs, and rhesus macaques before trial commencement. Validation in these animal models is not only time-consuming and costly but there is also a high technical barrier in undertaking *Mtb* challenge experiments in animals (requiring prolonged use of ABSL-3 level laboratories). In addition, the failure of the phase IIb clinical trial of the MVA85A vaccine showed that the currently available animal models do not predict human immunity well. Moreover, in the clinical trials evaluating TB vaccines, the assessment of protective efficacy relies on natural exposure to *Mtb* infection and requires enrollment and follow-up of tens of thousands of people for multiple years. The limited availability of suitable human populations in which this can be undertaken and the enormous cost of clinical trials further hinder the progress of TB vaccine development.

In recent years, the concept of a controlled human infection model has come under consideration to facilitate vaccine research progress. In this model, healthy volunteers are vaccinated with candidate vaccines and then are deliberately infected with the corresponding pathogen. The efficacy of the candidate vaccine is then assessed by either presence or absence of established infection or by disease progress. Recently, Vaxchora, a Cholera vaccine, was approved based on a human challenge study (122). In the field of TB, deliberately infecting healthy volunteers with virulent *Mtb* would not be ethical, since there is currently no method of anti-TB treatment that could reliably completely eradicate infection. As an alternative, Helen McShane, a co-inventor of MVA85A, described in 2012 a model in which humans are challenged with BCG (123). In this model, BCG-naive and BCG-vaccinated healthy volunteers in the UK were challenged with intradermal BCG, and the bacterial load was quantified from punch biopsies by PCR and bacterial culture (123). This model was used to assess the protective effect of MVA85A on BCG-vaccinated healthy adults (124), the data support the contention that this intradermal BCG challenge model is able to detect differences in anti-mycobacterial immunity induced by vaccination. In addition, an aerosol BCG challenge study is now underway in healthy UK adults to mimic the natural route of exposure (NCT02709278, NCT03912207).

Recently, Sarah Fortune and Eric Rubin described the development of an *Mtb* human challenge model, in which, *Mtb*’s growth is controlled by dependence on the availability of selection compounds; the bacteria are no longer viable once those compounds are removed. The study has not yet been published but was described in a commentary paper (125). This human *Mtb* infection model could substantially reduce the numbers of participants, study duration, and economic costs in TB vaccine studies.

### 4.2 Ultra-Low-Dose Aerosol Murine and Non-human Primate Infection Models

In general, in pre-clinical TB vaccine efficacy evaluation, animals are always infected using a single large bolus of *Mtb* that is delivered to the lungs by intratracheal or aerosol installation. However, in natural infection most people are infected by repeated inhalation of low doses. In animal models, the evidence for the efficacy of a TB vaccine is usually accepted as significant by the demonstration of 0.5-1 log_10_ lower numbers of *Mtb* in vaccinated compared to control animals at some point after challenge infection. A wide range of experimental and cost constraints dictate that the demonstration of larger and more meaningful effects with smaller infection challenges is difficult to achieve. Consequently, vaccine development often moves forward on the basis of modest vaccine impact compared to the potency required in human clinical efficacy testing, in which at least a 60% improvement in efficacy in prevention of the disease compared to BCG alone is required (126). These divergences between laboratory models and clinical scenarios hinder TB vaccine development.

Optimization of *Mtb* challenge doses in pre-clinical TB vaccine evaluation was undertaken in non-human primate models. Recent studies tended to use a lower dose than prior vaccine studies. For instance, in 2018, in the RhCMV/TB vaccine study, the monkeys received 10 or 25 bacteria (99). In 2019, a repeated limiting-dose challenge model used an average of 1.3 bacteria implanted weekly for 8 consecutive weeks by endobronchial installation (127). In 2020, in the BCG immunization route optimization study, the monkeys were challenged by bronchoscope with 4-36 bacteria (18). These low-dose-infection non-human primate models better mimicked the natural course of TB infection in humans and allowed investigators to observe vaccine-mediated sterilizing immunity.

In 2021, a model of ultra-low-dose aerosol infection in mice was established, in which infection was initiated by only 1-3 founding bacteria, instead of the conventional ~100 CFU dose. As in human TB, highly heterogeneous bacterial burdens, immune responses, and disease manifestations were observed in this model (128). In addition, the well-circumscribed granulomas shared features with human granulomas. Thus, this ultra-low dose infection murine model more closely replicates human disease. It is also much cheaper and easier to handle than the low-dose non-human primate models, thus, it might facilitate preclinical testing of vaccine and immunotherapeutic candidates and act as a gatekeeper to determine which vaccines show promise and warrant further testing.

### 4.3 T_RM_-Mediated Anti-TB Immune Protection

T_RM_ represents a distinct subset of memory T cells that was found in the past decade. Unlike other memory T cells such as central memory and effector memory T cells, T_RM_ cells colonize local tissues infected by pathogens and remain there for a long time after the pathogen has been eliminated without participating in blood circulation (129). They have been demonstrated at sites that include the intestines, skin, urogenital tract, and lung mucosa (130–133). When the pathogen invades again, T_RM_ cells immediately sense and initiate immune responses so that the pathogen can be controlled or be eliminated at the early stage of infection. This process does not depend on memory cells in the peripheral circulation and is in the first line of defense of the body as an adaptive immune response to pathogen infection (134). In 2014, Daniel L. Barber’s group reported for the first time the role of lung T_RM_ in anti-TB infection in a murine model. The *Mtb*-specific CD4^+^ T cells in lung tissues could be divided into two populations, namely, a subpopulation of T_RM_ colonizing the lung parenchyma and other memory cell subsets circulating in the vasculature; the former were identified with molecular markers as KLRG1^-^CXCR3^+^ and the latter as KLRG1^+^CX3CR1^+^ (135). The adoptive transfer of lung *Mtb*-specific T_RM_ between mice resulted in potent immune protection (135–137). Based on these observations, our investigation of the properties of circulating CD4+ T cells in patients with active TB led us to suggest that inhibition of KLRG1^+^ expression through the incorporation of a specific inhibitor of the Akt signaling pathway in a vaccine could enhance the protective responses in immunotherapeutic and perhaps prophylactic vaccination regimens (138).

In 2016, Stefan H. E. Kaufmann’s group reported for the first time T_RM_-mediated immune protection against TB infection, which was induced by mucosal delivery of BCG (139) and we reported that SeV85AB induced high levels of lung CD8^+^ T_RM_ by using a comprehensive intravascular staining method (42). In the same year, several TB vaccines were also reported to be able to induce T_RM_ (140–142). In fact, as early as 2010, researchers had found that treatment with FTY720 (an immunosuppressant that blocks memory cells in circulation (143)) partially counteracted the immune protection induced by the BCG vaccine (144), indirectly demonstrating that BCG possesses the ability to induce a certain level of T_RM_-mediated immune protection. In 2020, by using an intravascular staining method in non-human primates, intravenous BCG was shown to induce higher levels of lung parenchymal CD4^+^ T cells compared with intradermal vaccination, and this was associated with sterilizing immunity against *Mtb* challenge (18), indicating that vaccine-induced T_RM_ also conferred *Mtb* resistance in this model.

By mimicking the route of infection in vaccination, a mucosal or intravenous vaccination might be an optimal vaccination strategy, targeting the induction of immune responses at the point of entry of the bacteria. However, the role of T_RM_ in TB protection awaits further experimental confirmation; a better understanding of vaccine-induced lung T_RM_ would facilitate novel T_RM_-targeting vaccine designs.

### 4.4 Role of Trained Immunity in TB Vaccine Development

Traditionally, vaccine development is mainly focused on the induction of the adaptive immune response that elicits antigen-specific long-term immune memory against infection. However, recently it has been shown that innate immunity also plays an important role in immune memory against homologous or even heterologous challenges (145, 146). Trained immunity, a *de facto* innate immune memory, has been defined as a long-term functional reprogramming of the innate immune cells that is evoked by endogenous or exogenous insults, with the cells then returning to a non-activated state and showing altered inflammatory responses against a second challenge (147, 148).

In 2020, a randomized clinical trial of BCG vaccination in the elderly showed immune protection against heterologous infections and improved survival (149). In 2021, another investigator-blind randomized controlled trial showed that BCG vaccination at birth significantly reduced all-cause infectious disease morbidity during the neonatal period (150). Trained immunity was proposed to be implicated in such BCG-induced heterologous protection. The first report of BCG-induced trained immunity showed that BCG vaccination in healthy volunteers enhanced the release of monocyte-derived cytokines in response to unrelated bacterial and fungal pathogens, and induced lymphocyte–independent protection of immunodeficiency SCID mice against disseminated candidiasis (151). In a study of BCG-induced trained immunity against *Mtb* infection in people, a global DNA methylation analysis revealed a stable and robust differential DNA methylation pattern among the promoters of genes belonging to immune pathways in “responders” to BCG vaccination but not in non-responders. Responders were defined as having an enhanced macrophage capacity to restrict the growth of *Mtb* associated with higher levels of IL-1β production (152). In rhesus macaques, mucosal or intravenous BCG inoculation conferred better protection against *Mtb* infection and TB disease than standard intradermal vaccination, and this was associated with the induction of enhanced trained immunity (153). β-glucan-induced trained immunity also afforded protection against *Mtb* infection (154). In contrast to BCG, *Mtb* infection impairs the development of protective trained immunity through impacting IFN-I signaling (155). These data suggest that vaccines that are aimed at enhancing trained immunity might give better protection against *Mtb* infection.

In 2018, the inventors of AdAg85A reported that respiratory infection with adenovirus could induce alveolar macrophages (AMs) that had a long-lasting memory that was sustained by an enhanced trained immunity phenotype in the local mucosal sites (156). This study suggested that non-specific trained immunity induced by the virus-vectored TB vaccine might contribute to the immune protection against *Mtb* infection. In 2020, they used a murine model of TB vaccination to investigate the role of AMs in host defense against *Mtb* and showed that respiratory mucosal immunization with AdA85A provided a type of trained immunity capable of potent protection against *Mtb* in the early stage of infection (157). In 2022, they further showed that mucosal immunization is superior to intramuscular immunization for the induction of trained immunity in AMs in a murine model of the SARS-CoV-2 vaccine (158), further adding to the evidence for the importance of local induction of trained immunity.

Cumulatively, the evidence suggests that the deciding battleground is the apoptosis of *Mtb*-infected macrophages in early infection, which is mediated by AMs and is enhanced by trained immunity. However, our understanding of the relative contribution of trained immunity to viral-vectored vaccine induction of T cell-mediated immune protection against TB remains limited. In addition, it remains to be answered that whether the anti-TB immune protection induced by BCG-prime-viral vector boost strategy is associated with the trained immunity, and the effective of live viral vector boost on innate immune training by BCG prime. Unraveling the elaborate molecular mechanisms of trained immunity will be critical for devising novel approaches to optimize the exploitation of trained immunity by TB vaccines.

### 4.5 Lack of Validated Immune Correlates of Protection by TB Vaccines

COPs are defined as laboratory biomarkers that are associated with protection from clinical disease. In particular, vaccine-inducible COPs are expected to be transformative in developing novel vaccines as they will de-risk the selection of candidate vaccines for human efficacy studies at an early stage. They might also substantially reduce the costs of large-scale clinical trials by helping to tailor the selection of participants being enrolled and by measuring vaccine immunogenicity and potential efficacy as a supplement, or even sometimes an alternative, to assessments of disease burden (159). Once validated in efficacy trials, COPs could potentially facilitate the development and licensure of vaccines. The absence of reliable parameters that could be used as COPs for TB vaccines represents one of the greatest challenges in TB vaccine development.

The complexities of *Mtb* infection create challenges in finding predictive markers of protective efficacy. The Th1 cytokines IFN-γ, IL-2, TNF-α, the Th17 cytokine IL-17, and other cytokines are active in the immune response against *Mtb* and are used as biomarkers to determine the antigen-specific T cell responses in TB vaccine evaluation research. However, studies have found that IFN-γ accounted for only about 30% of the CD4^+^ T cell-mediated immune protection against *Mtb* infection; and its overexpression even accelerated death in infected mice (137). More recently, close contacts of active TB patients who were persistently negative by IFN-γ release assay and tuberculin skin tests were defined as “resisters” of *Mtb* (160) and in 2019, a cohort study showed that “resisters” possess IgM and class-switched IgG antibody responses and non-IFN-γ T cell responses to *Mtb*-specific proteins (161), challenging the rationality of focus on assessing IFN-γ-based immunogenicity in TB vaccine design. In 2020, we found that T-cell activation status marker CD69 is associated with *Mtb* infection and may have the potential to distinguish latent TB infection (positive IFN-γ responses) and “resisters” (negative IFN-γ responses) (162). Based on these and similar studies, there is an urgent need to find novel molecular markers that are more correlated with immunogenicity/protective efficacy to be able to more accurately predict the protective efficacy of vaccines and to accelerate vaccine evaluation.

As mentioned above, T_RM_ showed a potential for application among novel COPs in TB vaccine research. However, validation of vaccine-induced COPs is possible only when successful placebo-controlled efficacy trials become available. Only then can compelling comparisons be made. In 2019, a phase IIb clinical trial of the subunit TB vaccine M72/AS01_E_ showed 49.7% efficacy against progression to TB compared with placebo control. This is the first novel TB vaccine to almost reach 50% protection in the past century (163). These vaccine cohorts offered an opportunity to identify COPs of vaccine-induced immune protection against *Mtb* infection and some strategies are in place. Bill & Melinda Gates Medical Research Institute, vaccine manufacturers, sponsors of clinical trials, and trial investigators have launched an international “TB Immune Correlate Program” consortium to identify immunological COPs for TB. The first priority is informed by existing knowledge and recent findings from animal models and clinical studies, including the magnitude of mycobacteria-specific Th1/Th17 CD4 T cell responses, magnitude/subclass/avidity of mycobacteria-specific mucosal IgA or IgG antibody responses, Fc-mediated, functional antibody activities, and trained immunity (27). However, the relatively small number of participants that reached clinical endpoints in the M72/AS01_E_ clinical trial might restrict the statistical power of COPs discovery. Thus, larger clinical trials or the human infection model study are needed to validate the COPs that might be identified in the ongoing efforts.

## 5 Conclusion

Identified at the end of 2019, COVID-19 became a global public health threat within 3 months, it spread over the globe so rapidly that it was declared to be a “pandemic” by the WHO in March, 2020. Vaccinologists worked on the challenge immediately, leading to the development and deployment of novel vaccines within one year. Up to February of 2022, 144 and 195 COVID-19 vaccine candidates based on diverse platform technologies are being evaluated in clinical and preclinical stages, respectively, and dozens of vaccines have already been licensed to human use (164). The rapid COVID-19 vaccine development and deployment is critical for the world to return to pre-pandemic normalcy. Ironically, the only licensed TB vaccine is still the one-century-old BCG, which is inadequate. Thus, TB remains a leading cause of mortality from an infectious disease, only now surpassed by SARS-CoV-2 causing COVID-19. Considering the morbidity and mortality that is suffered from TB globally, it is time to accelerate commitment, investment, and implementation to stop the infectious disease agent that has killed more human beings than any other.

We believe that the success of COVID-19 vaccines and recent progress in TB vaccine research illustrate that the deployment of an effective TB vaccine is likely in the near future. The highly efficacious COVID-19 vaccines accelerated the vaccine development process in human use, with the notable example of mRNA vaccines and adenovirus-vectored vaccines, and increased the public acceptance of the concept of the controlled human challenge model, which might provide valuable experience on the development of TB vaccines. In this review, we have provided an update on the current viral vectored TB vaccine pipeline and summarized the latest findings that might facilitate TB vaccine developments. On the one hand, several viral vectored TB vaccines are in clinical trials, and other promising candidate vaccines at an earlier stage of development are being evaluated in preclinical animal models, and this sharply increases the likelihood of developing an effective TB vaccine in the near future, although this is far from certain. On the other hand, we propose that a better understanding of the lung-resident T_RM_-mediated mucosal immunity, and the unique trained immunity of phagocytic cells against intracellular *Mtb* infection, could help provide novel targets for innovative and superior TB vaccine designs. Moreover, new tools, such as controlled human infection and ultra-low-dose aerosol infection murine infection models, should facilitate TB vaccine development and selection in the preclinical phase of the investigation. In addition, identification of COPs in the M72/AS01_E_ trial and other ongoing clinical trials could be valuable in streamlining triage and evaluation of next-generation TB vaccine candidates. Allocation of resources must include the discovery and development of early pipeline candidates to increase clinical trial capacity. With more advanced knowledge, we remain hopeful that a more effective TB vaccine will be developed sooner rather than later.

## Author Contributions

ZH and X-YF conceived, designed and wrote the manuscript, X-YF, S-HL and DL edited the manuscript with conceptual advice. All authors contributed to the article and approved the submitted version.

## Funding

This work was supported by National Key Research and Development Program of China (2021YFC2301503), Grants from the National Natural and Science Foundation of China (81873884, 82171815, 82171739), Shanghai Science and Technology Commission (19XD1403100, 20Y11903400), and Shanghai Municipal Medical and Health Excellent Young Talents Training Program (GWV-10.2-YQ01).

## Conflict of Interest

The authors declare that the research was conducted in the absence of any commercial or financial relationships that could be construed as a potential conflict of interest.

## Publisher’s Note

All claims expressed in this article are solely those of the authors and do not necessarily represent those of their affiliated organizations, or those of the publisher, the editors and the reviewers. Any product that may be evaluated in this article, or claim that may be made by its manufacturer, is not guaranteed or endorsed by the publisher.
